# Impaired Lumbar Extensor Force Control Is Associated with Increased Lifting Knee Velocity in People with Chronic Low-Back Pain

**DOI:** 10.3390/s23218855

**Published:** 2023-10-31

**Authors:** Adrian Pranata, Joshua Farragher, Luke Perraton, Doa El-Ansary, Ross Clark, Denny Meyer, Jia Han, Benjamin Mentiplay, Adam L. Bryant

**Affiliations:** 1 School of Health and Biomedical Science, RMIT University, Mill Park 3082, Australia; joshua.farragher@rmit.edu.au (J.F.); doa.el-ansary@rmit.edu.au (D.E.-A.); 2 School of Science, Computing and Engineering Technologies, Swinburne University of Technology, Hawthorn 3122, Australia; 3 College of Rehabilitation Sciences, Shanghai University of Medicine and Health Sciences, Shanghai 201318, China; jia.han@canberra.edu.au; 4 School of Exercise and Health, Shanghai University of Sport, Shanghai 200438, China; 5 Centre for Health, Exercise and Sports Medicine, The University of Melbourne, Parkville 3052, Australia; albryant@unimelb.edu.au; 6 Department of Physiotherapy, Monash University, Frankston 3199, Australia; luke.perraton@monash.edu; 7 Department of Surgery, Royal Melbourne Hospital, Parkville 3052, Australia; 8 School of Health, University of Sunshine Coast, Sippy Downs 4556, Australia; rclark@usc.edu.au; 9 School of Health Sciences, Swinburne University of Technology, Hawthorn 3122, Australia; dmeyer@swin.edu.au; 10 Research Institute for Sports and Exercise, University of Canberra, Bruce 2617, Australia; 11 LaTrobe Sport and Exercise Medicine Research Centre, La Trobe University, Bundoora 3086, Australia; b.mentiplay@latrobe.edu.au

**Keywords:** low-back pain, kinematics, lifting, force control, motor control, correlation

## Abstract

The ability of the lumbar extensor muscles to accurately control static and dynamic forces is important during daily activities such as lifting. Lumbar extensor force control is impaired in low-back pain patients and may therefore explain the variances in lifting kinematics. Thirty-three chronic low-back pain participants were instructed to lift weight using a self-selected technique. Participants also performed an isometric lumbar extension task where they increased and decreased their lumbar extensor force output to match a variable target force within 20–50% lumbar extensor maximal voluntary contraction. Lifting trunk and lower limb range of motion and angular velocity variables derived from phase plane analysis in all planes were calculated. Lumbar extensor force control was analyzed by calculating the Root-Mean-Square Error (RMSE) between the participants’ force and the target force during the increasing (RMSE_A_), decreasing (RMSE_D_) force portions and for the overall force error (RMSE_T_) of the test. The relationship between lifting kinematics and RMSE variables was analyzed using multiple linear regression. Knee angular velocity in the sagittal and coronal planes were positively associated with RMSE_A_ (R^2^ = 0.10, β = 0.35, *p* = 0.046 and R^2^ = 0.21, β = 0.48, *p* = 0.004, respectively). Impaired lumbar extensor force control is associated with increased multiplanar knee movement velocity during lifting. The study findings suggest a potential relationship between lumbar and lower limb neuromuscular function in people with chronic low-back pain.

## 1. Introduction

The lumbar extensor (LE) muscle group is comprised of the multifidi, erector spinae and short and intersegmental muscles and is involved in controlling lumbar posture and movements [1]. The LE muscle group is capable of generating forces directed in the sagittal, frontal and axial planes, resulting in compression and posterior shear forces on the spine [2]. The neuromuscular function of the LE muscle group has been demonstrated to be altered in people with chronic low-back pain (CLBP) [3], the biggest contributor to disability in industrialized countries [4]. In the literature, people with CLBP typically present clinically with pain across the lower back region between L1-S1 spinal segments, with or without associated unilateral or bilateral leg pain [5], have demonstrated variable trunk muscle activation patterns [3], delayed lumbar muscle activation to perturbation [6] and impaired lumbar proprioception, particularly in sitting [7] compared to healthy controls. Additionally when compared to healthy controls, people with CLBP also demonstrated impairment in sub-maximal LE muscle force control [8].

Lumbar extensor muscle force control is defined as the ability of the LE muscle group to produce accurate force [8]. LE muscle force control is typically assessed by assessing isometric LE muscle force steadiness [9] and isometric muscle force accuracy using a moving—i.e., variable force target [8]. Force accuracy is typically quantified by calculating the root-mean-square error between the participants’ force output and target force [8]. Pranata et al. [8] demonstrated that people with CLBP exhibited 30–45% more LE force matching error (i.e., overshot or undershot the target force)—hence, they exhibited decreased force control compared to healthy controls. Additionally, the inability to produce accurate force has been demonstrated to be associated with increased CLBP-related disability, suggesting the clinical relevance of the novel LE muscle force control assessment [8].

The function of the LE muscle group in people with CLBP can also be evaluated, in part, by assessing the kinematics of functional movements such as lifting. Lifting is an activity of daily living that is usually performed at submaximal intensities and requires an appropriate level of muscle force control for coordinated movement of the trunk and lower limb. Lifting-related kinematic strategies are known to vary between CLBP and healthy individuals, with high variability demonstrated in the in those with CLBP [10] which are task dependent [11]. For instance compared to healthy controls, decreased inter-subject trunk movement variability has been observed during trunk flexion-extension tasks performed at self-selected pace [12]. Similarly, decreased hip and knee coordination variability was observed during a free-style lifting task in people with CLBP when compared to healthy controls [13].

It has been proposed that CLBP could be associated with adverse changes distal to the trunk—such as in the hip and knee during functional task performance [14]. Recent studies have reported decreased in hip abductor [15] and quadriceps [16] strength in people with CLBP which could affect functional task performance. Furthermore, recent studies have demonstrated that ankle proprioception could be impaired in people with CLBP [17]. People with CLBP have demonstrated poorer standing balance that is reflected in increased postural sway during quiet standing [18,19]. That said, it is unknown whether dynamic tasks, such as lifting, is affected by trunk and lower limb neuromuscular impairments in people with CLBP. However, overall, the research in this area is sparse.

This study proposed that impairments in LE muscle force control could contribute to changes in lower limb kinematics (e.g., increased lower limb movements) which in turn could alter dynamic posture and subsequently task performance such as lifting in people with CLBP. Thus, the aim of this study is to investigate the relationship between LE muscle force control and lower limb kinematics during lifting in the sagittal and coronal planes. It was hypothesized (H_1_) that there would be a significant (positive or negative) association between LE muscle force control (i.e., target matching error) and lower limb lifting range of motion (ROM) and angular velocity. A null hypothesis (H_0_) of this study was there would be no significant association between LE extensor muscle force control and lifting lower limb kinematic variables.

## 2. Materials and Methods

### 2.1. Participants

A pragmatic sample of thirty-three participants (n_female_ = 18, n_male_ = 15) aged 25–60 years with CLBP were recruited from a large physiotherapy clinic in Melbourne, Australia. Participants were new patients of the clinic who reported pain between the level of the twelfth thoracic vertebra (T12) and the gluteal fold >3 months. Physiotherapists screened and excluded participants if they presented with overt neurological signs such as muscle weakness associated with lumbar radiculopathy or myelopathy, previous spinal surgery, systemic or inflammatory conditions, malignancy, unstable spondylolisthesis (i.e., specific diagnosis of CLBP) using a physical assessment framework previously described [20] or inability to understand written or spoken English. Ethics approval was obtained from The University of Melbourne’s Behavioural and Social Sciences Human Ethics Committee (ID: 1340715). All participants provided written informed consent prior to entering the study.

### 2.2. Outcome Measures and Experimental Procedure

#### 2.2.1. Pain and Disability

All participants completed the widely used and validated Oswestry Disability Index (ODI) a measure of CLBP-related disability [21] and rated their pain out of 10 using a Numerical Rating Scale [22] prior to commencing the laboratory session.

#### 2.2.2. Lumbar Extensor Muscle Force Control

Details of the experimental protocol for assessing LE muscle force control have been described in detail previously [8]. Isometric LE strength was derived from the assessment of LE muscle maximum voluntary isometric contraction (MVIC). Maximum voluntary isometric contraction of the LE muscle was performed by instructing the participants to push against the backrest as hard as possible whilst seated on the MedX (Ocala, FL, USA) LE dynamometer. The MedX is a valid [23] and reliable (r = 0.57–0.93, SEE = 12.0–44.5 Nm) [24] instrument to measure LE strength in people with CLBP.

**Figure 1:**
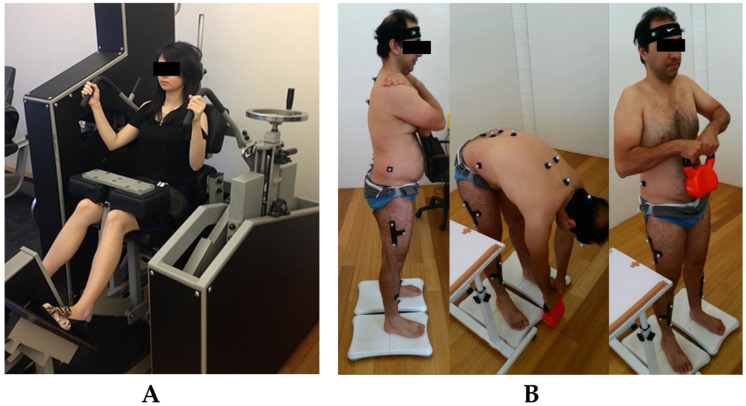
Testing procedures of LE muscle force control (**A**) and lifting kinematics (**B**).

For the assessment of submaximal LE muscle force control, participants were seated in a lumbar dynamometer machine and locked in 12° lumbar flexion (i.e., upright sitting, 0° is full extension). Using visual biofeedback displayed on a tablet computer placed 1 m in front of the participants, participants were instructed to press their back against the backrest, increasing and decreasing isometric force output to match a variable force target that moves at a frequency of 0.08 Hz (Figure 1A). Participants were instructed to match a moving force target that varies between 20% MVIC (lower force limit) and 50% MVIC (upper force limit) as accurately as possible by increasing and decreasing LE isometric force production over a 1 min period. No verbal encouragement was provided, and the environment was kept silent. Prior to the data collection proper, participants were provided with one practice trial and a 30 s rest. As such, participants were required to complete ~5 ascending and ~5 descending cycles of a sinusoidal wave.

#### 2.2.3. Lifting Kinematic Assessment

Likewise, the methodology for the lifting kinematic assessment has been described in detail elsewhere [13]. Twenty-one retro-reflective markers of 13 mm diameter were attached to pre-specified anatomical landmarks using a double-sided tape to create a thorax, pelvis, thigh and shank segments. Thoracic marker configuration was similar to previously published study by Christe et al. [25] as such that the lumbar movement is the rotational movement between the thorax and pelvis segments. Hip (pelvis-thigh) and knee (thigh-shank) joints were derived using the longitudinal axes of each segment. Participants stood in front of a 12-camera Optitrack Flex 13 motion capture system (NaturalPoint, Corvallis, OR, USA). They were instructed to lift an 8 kg kettlebell up to the level of their abdomen using a self-selected technique and pace (Figure 1B). The 8 kg weight was selected as this was the average weight of a bag of groceries [26]. The movement was repeated twice, the first served as a practice trial.

### 2.3. Data Analysis

**Figure 2:**
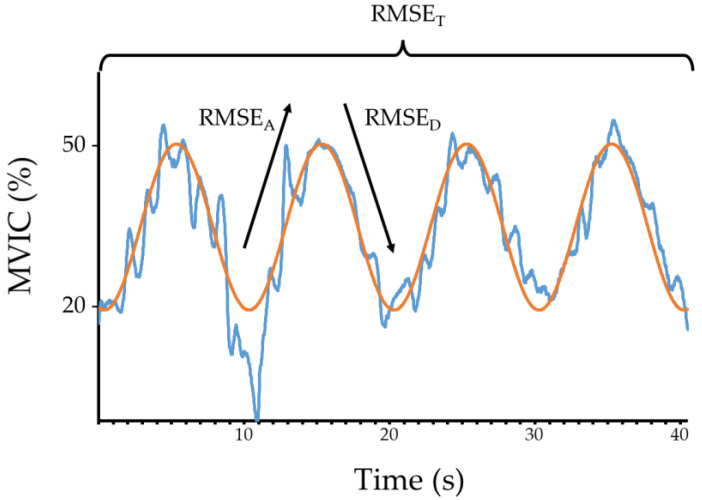
An analysis of LE muscle force control assessment for a CLBP participant. Orange trace = target force, blue trace = participant’s force. RMSE_A_ = average root-mean-squared error during ascending/ramp-up phase of the test, RMSE_D_ = average root-mean-squared error during descending/ramp-down phase of the test, RMSE_T_ = average total root-mean-squared error during testing.

Lumbar extensor isometric strength data from the MedX was filtered using a low-pass Symlet-8 undecimated wavelet filter with a frequency of 62.5 Hz [8] and converted to torque in Newton meters (Nm) using a custom-written LabVIEW software (National Instruments, Austin, TX, USA) [8]. The custom data acquisition system was calibrated by applying a series of loads to the MedX dynamometer, recording the results from the MedX software and raw data from the data acquisition system and creating a calibration factor for the raw data with the MedX results as the criterion reference using linear regression analysis. Lumbar extensor muscle force control was quantified using the root-mean-squared error (RMSE) between the participant’s torque output and the target torque. RMSE was calculated for the ascending or ramping up phase (i.e., the force error between 20–50% MVIC; RMSE_A_), descending or ramping down phase (i.e., the force error between 50–20% MVIC; RMSE_B_) and average total error (RMSE_T_) [8]. This resulted in five ascending and five descending cycles during data collection. For data analysis, the first and last waveforms were removed, resulting in four ascending and four descending cycles (Figure 2). Kinematic data was cleaned, and gap filled using the Optitrack Motive software (NaturalPoint, Corvallis, OR, USA) and passed through a custom written kinematic data analysis LabVIEW pipeline (National Instruments, Austin, TX, USA). Joint angle data, in degrees, were filtered using a fourth order zero-phase shift low-pass Butterworth filter with a 6 Hz cut-off frequency [27]. The start position for the lifting task was the position where the lumbar spine was at its maximum flexion. The end lifting position was where the lumbar position was at its maximal extension (i.e., upright position). The kinematic variables between sides were averaged. Following this, the average ROM and angular velocity (first derivative of angular displacement) of the lumbar, hip and knee joints in the sagittal (*x*-axis) and coronal (*y*-axis) planes were obtained for statistical analyses.

### 2.4. Statistical Analysis

Linearity and strength of relationships between the independent variables (i.e., lumbar, hip and knee ROM and VEL in *x* and *y*-axes) and dependent variable (i.e., RMSE variables) were analyzed using Pearson product-moment correlation coefficient and scatterplots. Normality, homoscedasticity and linearity of the residuals of the regression analyses were assessed using Levene’s test and scatter graphs. Kinematic variables that exhibited a significant correlation with RMSE variables were included in a series of multivariate linear regression models. All analyses were conducted with significance level set at 0.05 using SPSS Version 21.0 (IBM, Inc., Chicago, IL, USA).

## 3. Results

Descriptive data pertaining to participant characteristics are presented in Table 1. Participant LE muscle force control and lifting kinematic variables are presented in Table 2 and Table 3, respectively. Only knee angular velocity variables were positively associated with the RMSE variables. Specifically, RMSE_A_ was positively correlated with knee angular velocity in the sagittal (r = 0.35, *p* = 0.046) and coronal (r = 0.48, *p* = 0.004) planes. RMSE_D_ was positively correlated with knee angular velocity in the coronal (r = 0.37, *p* = 0.034) plane. RMSE_T_ was positively correlated with knee angular velocity in the coronal plane (r = 0.36, *p* = 0.039). There was no significant correlation between the lumbar and hip kinematic variables and the RMSE variables.

**Figure 3:**
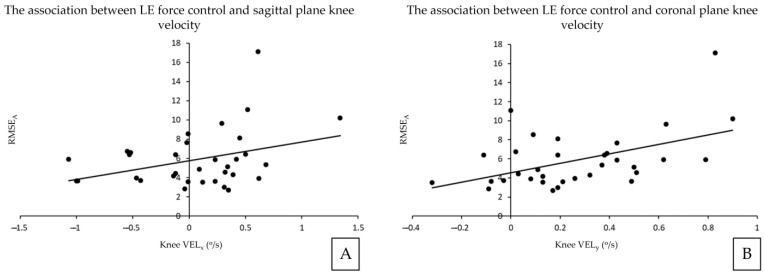
The associations between lumbar extensor force control and lifting parameters. RMSE_A_ and Knee VEL_x_ (**A**), RMSE_A_ and Knee VEL_y_ (**B**). RMSE_A_ = average root-mean-squared error during ascending phase, Knee Vel_x_ = knee angular velocity in the sagittal plane movement, Knee Vel_y_ = knee angular velocity in the coronal plane movement.

Results of the linear regression analyses between kinematic and RMSE variables are summarized in Figure 3. Knee angular velocity in the sagittal plane was positively associated with RMSE_A_ (adjusted R^2^ = 0.10, β = 0.35, *p* = 0.046). Knee angular velocity in the coronal plane was also positively associated with RMSE_A_ (adjusted R^2^ = 0.21, β = 0.48, *p* = 0.004).

## 4. Discussion

The aim of this study was to investigate the relationship between LE muscle force control and lifting-related lumbar and lower limb movement variables in the sagittal and coronal planes. To our knowledge, this is the first published study to investigate the relationship between LE force control and lifting kinematics. A previous study has demonstrated that LE submaximal muscle force control is impaired in people with CLBP when compared to healthy controls [8]. In particular, people with CLBP demonstrated 30% less ability to produce LE accurate force when compared to healthy controls [8]. The result of this study adds to the previous research and indicates that the inability to produce accurate LE force is associated with increased knee movements during lifting. This means, people with CLBP with poorer LE force control compensated more with their legs during lifting, evident by increasing knee movements during lifting. 

The study findings supported our hypothesis that LE force control is associated with changes in lifting lower limb kinematics. Specifically, LE force control partially explains the variances in knee angular velocity in the sagittal and coronal planes. CLBP-related impairments in trunk muscle function are well described in the literature and include: increased trunk muscle co-contraction [3], delayed trunk muscle response to perturbation [28], altered trunk and lower limb movement coordination [13,29]. Increased trunk stiffness via trunk muscle co-contraction [30] has been identified as a strategy to maintain spinal posture during dynamic tasks (e.g., lifting) in people with CLBP [31]. However, excessive trunk stiffness [32]—which is strongly associated with imprecise force production, can potentially impair dynamic task performance as a result of delayed activation of the trunk and lower limb muscles resulting in higher joint excursion and postural sway [33,34] leading to a potential loss of balance [35]. Increasing trunk muscle activation (i.e., stiffness) could be implemented by people with CLBP as a preferred strategy to decrease their dependence on cognitive feedback mechanism during complex task performance [36] such as lifting at the expense of maintaining dynamic balance.

Movements at the hips and knees in all planes have been proposed to improve balance during dynamic task performance by aligning the body center-of-gravity within the base of support. This motor behaviour may have been reflected in this study findings. Our findings are also in line with Mitchell et al. who observed increased lower limb movements, knee movements in particular, during a step-up task in people with CLBP when compared to healthy controls [37]. Interestingly, Mitchell et al. [37] found that during step-up movement—a task that is predominantly performed in a sagittal plane, people with CLBP demonstrated increased knee movements in the coronal plane (i.e., out of plane movement). Similarly, when compared to healthy controls, people with CLBP had the tendency to utilize more hip and knee movements, in reference to the ankle, during a deep squat task to achieve similar maximum squat depth as healthy controls [38]. The dynamic task utilized in this study, bilateral lifting, also predominantly required movements of the lumbar, hip and knee regions in the sagittal plane. The requirement for additional stability in the lower limb is also evident by reports of decreased hip and knee movement variability in people with CLBP compared to healthy controls during lifting [13]. The reason why people with CLBP preferred out of plane movements in the knee (i.e., in the coronal plane) was not clearly explained in the literature. These findings could be explained by the concept of Regional Interdependence, where a patient’s primary musculoskeletal symptoms may be directly or indirectly influenced by other body regions or systems, regardless of the proximity of the primary symptoms [39]. In this case, people with CLBP presenting with compromised trunk muscle function could be more reliant on their lower limbs to perform a dynamic task such as lifting.

Lifting is a task that requires activation of the LE muscle group and lower limb muscles (e.g., hamstring, quadriceps) [40] to initiate and control lumbar and lower limb extension movement. In this study, most participants stoop lifted (i.e., lifting with the knee relatively straight; average participant knee ROM was 13.7°) to lift the load off the ground. When utilizing a stooped lifting technique, the lumbar and hip joint movements contribute to power generation whilst the knee flexor moment and the co-contraction of lower limb antagonistic muscles (i.e., the quadriceps and hamstrings; the anterior tibial compartment muscles and the calf muscles) appear to be critical to attenuate forces imposed on the lumbar joints [41,42]. Specifically, forces generated through the lower limbs may be transmitted to the spine through the thoracolumbar fascia that has fibers spanning from the occiput to the sacrum, erector spinae aponeurosis and sacrotuberous ligament [43]. Thus, the knee movements observed in CLBP participants may not provide a stable base (i.e., constantly moving center-of-gravity about the base of support) required for optimum force transmission up the kinetic chain which may, in turn, impact accurate force production in the lumbar spine. Furthermore, lower limb-related impairments (e.g., decreased strength and endurance of gluteus maximus and gluteus medius [44,45], decreased quadriceps strength [16,44] and decreased hamstring length [46]) have also been observed in people with CLBP when compared to healthy controls, and these may adversely affect lower limb force-generating and attenuating capacities. As it is the role of the trunk to position the peripheral joints optimally for functional tasks, impaired force control may also alter the lower limb kinetic chain synchrony during lifting, which may, in turn, adversely affect LE muscle force control.

Results of this study suggest a link between knee movement during lifting and LE muscle force control which in turn, could be associated with disability (i.e., decreased LE muscle force accuracy is associated with increased disability [8]). Furthermore, although not observed in this study, questions could be raised whether CLBP could predispose one to a knee injury. In a prospective study by Zazulak et al. [47], history of low-back pain has been demonstrated to increase the risk of knee injuries (e.g., anterior cruciate ligament injuries) in athletes. CLBP can have prolonged adverse impact on trunk neuromuscular control—even after pain has subsided [3] which may impact on long-term lower limb health. More recent study [48] also suggests that better trunk neuromuscular control (e.g., the ability to decrease the amount of lateral trunk lean) during fast cutting movements during sporting performance could decrease the risk of anterior cruciate ligament injuries. Furthermore, training trunk neuromuscular control (e.g., ‘core stability’ training) has been demonstrated to decrease the risk of anterior cruciate ligament injury due to its impact on knee valgus angle (decreased), hip adduction angle (decreased) and vastus medialis and lateralis activation ratio (increased) in side-stepping cutting task [49]. These studies further provide a link between trunk and lower limb neuromuscular control in people with CLBP.

From this study findings, by association, it is tantalizing to postulate that training of lower limb neuromuscular function, in particular knee control may improve LE muscle force control in people with CLBP. However, no study to date has investigated the effects of lower limb training on LE muscle force control in people with CLBP. Similarly, neuromuscular retraining of LE muscle force control (e.g., targeted training of force matching ability of the LE muscles across varying submaximal target force) may be associated with improvements in lower limb kinematic performance during lifting in people with CLBP. Research in this area is emerging [50]. Thus, future studies should continue to investigate the effects of LE muscle force control training in people with CLBP on lifting performance.

There are several limitations associated with this pilot study. Firstly, the LE muscle force control test utilized in this study was quasi-isometric with the participants’ lumbar spine and lower limbs fixed in the dynamometer. In contrast, lifting is a dynamic task involving concentric and eccentric trunk muscle contraction. Moreover, our study participants mostly performed a stooped lifting technique (i.e., high amount of lumbar and hip movement) with minimal knee flexion akin to their LE muscle force control testing position on the dynamometer. Thus, perhaps this explains why only lower limb kinematics (i.e., the knee) were associated with LE muscle force control in this study. Therefore, future studies should assess trunk muscle force control (e.g., muscle force matching task) under dynamic conditions. At this point of writing, there was a dearth of evidence on how the assessment of dynamic LE muscle force control could be performed in a clinical or laboratory setting. This could be a focus for future research. Secondly, our frequency selection for the variable force target may not reflect lifting-related task demands (e.g., lifting weight or technique). Further research is required to investigate different variable force target frequencies for trunk force control assessment. Finally, this study utilized a small pragmatic sample size of thirty participants and thus, only three predictor variables could be included in the multivariate regression analyses. Therefore, this study could not take into account potentially important non-modifiable covariates, such as gender or duration of back pain [51]. However, ex post facto correlation analyses of these variables with the regression input variable were performed and they were not significantly correlated. This indicates that they are unlikely to have influenced our results. Furthermore, this study did not take into account the impact of pain-related fear (e.g., fear of movement) which could impact lifting performance. It has been established that a higher fear of movement is associated with a higher CLBP disability [52]. However, the participants in this study reported relatively low levels of pain (mean = 3.30 out of 10) and disability (mean ODI = 20.7% or minimal disability), indicating that participants could cope with most living activities including lifting. Therefore, it is unlikely that fear of movement would significantly impact the result of this study. Lastly, this study did not target the ankle region as part of the lower limb kinematic analysis. Ankle proprioception has been demonstrated in people with CLBP [17] which may have adverse implications on functional tasks, such as the possibility of increased postural sway during lifting. As indicated by the possibility of regional interdependence [39], trunk neuromuscular impairments could be associated with impaired ankle function. Thus, the relationship between ankle kinematics and trunk muscle force control should be explored in future studies. Due to the small number of participants associated with this pilot study, we could not account for potential gender differences associated with our lifting task (e.g., gender-associated differences in trunk and lower limb muscle strength that may impact lifting performance). Readers should also remember that given the cross-sectional nature of this study, it is important to acknowledge the possibility of reverse causation. Specifically, it is unclear whether neuromuscular adaptations explored in this study had existed prior to the onset of CLBP.

## 5. Conclusions

Decreased ability to produce accurate LE muscle force was associated with increased knee movement velocity in the sagittal and coronal planes during lifting in people with CLBP. People with CLBP presenting with compromised trunk muscle function may be more reliant on their lower limbs—in particular, the knee joint—to perform a lifting task. This explorative study suggests a potentially important relationship between the lower limb and trunk neuromuscular function in people with CLBP which should be investigated in future studies.

## Figures and Tables

**Table 1:** Descriptive data (mean (SD)) pertaining to CLBP participant characteristics.

Variables (Units)	Mean (SD)
Age (years)	41.8 (10.8)
Gender (female, %)	18 (54.5%)
Height (m)	1.70 (0.1)
Mass (kg)	75.3 (17.7)
BMI (m/kg^2^)	25.2 (4.7)
CLBP duration (months)	107.5 (119.0)
ODI (%)	20.7 (12.3)
NRS (/10)	3.30 (1.8)

Values indicate mean (standard deviation), n = number of participants, BMI = Body Mass Index, ODI = Oswestry Disability Index, NRS = Numerical Rating Scale.

**Table 2:** Descriptive data (mean (SD) pertaining to CLBP lumbar extensor muscle force control.

Variables	Mean (SD)
RMSE_A_	5.87 (2.96)
RMSE_D_	4.13 (1.35)
RMSE_T_	5.21 (1.91)

RMSE_A_ = average root-mean-squared error during ascending phase, RMSE_D_ = average root-mean-squared error during descending phase, RMSE_T_ = total average root-mean-squared error, SD = standard deviation.

**Table 3:** Descriptive data (mean (SD)) pertaining to CLBP lifting biomechanical parameters.

Variables		Body Parts	
		Hip	Knee
Range of motion (^o^)	X	34.06 (9.31)	13.27 (7.60)
	Y	3.71 (2.54)	3.43 (2.34)
Angular velocity (^o^/s)	X	13.89 (6.15)	5.53 (3.82)
	Y	1.52 (1.26)	1.44 (1.25)
Total lifting time (s)		2.71 (0.97)	

X = sagittal plane movement, Y = coronal plane movement.

## Data Availability

Data will be made available upon direct request to corresponding author.
